# Breathing Is Enough: For the Spread of Influenza Virus and SARS-CoV-2 by Breathing Only

**DOI:** 10.1089/jamp.2020.1616

**Published:** 2020-07-28

**Authors:** Gerhard Scheuch

**Affiliations:** GS Bio-Inhalation GmbH, Headquarters & Logistics, Gemuenden, Germany.

**Keywords:** exhaled aerosols, influenza, SARS-CoV-2, virus transmission

## Abstract

***Background:*** The transmission of respiratory viruses such as influenza and corona viruses from one person to another is still not fully understood.

***Methods:*** A literature search showed that there is a strong scientific rationale and evidence that viruses are very efficiently spread through aerosols by the patient's breathing only. It is not necessary for the patient to cough or sneeze.

***Results:*** The exhaled aerosol particles are generated by normal breathing in the deep lung through reopening of collapsed small airways during inspiration. These mucus/surfactant aerosols (size range between 0.2 and 0.6 μm) can transport viruses out of the lungs of patients and be present in the room air for hours.

***Conclusion:*** These aerosol particles are difficult to filter out of the air; because of their physical properties, new strategies must be developed to protect people from these virus aerosols.

## History

In 1986 and 1987, a team of aerosol researchers from the Institute for Biophysics at the GSF (Research Center for Environment and Health) in Frankfurt investigated inhaled and exhaled aerosol particles. Their goal was to measure the growth of these aerosol particles in the airways. Using a very powerful laser directly in front of the mouth, they were able to measure both the number and the size of inhaled and exhaled particles.^(1)^ With a two mode laser photometer, they were able to count and measure aerosol particles in a size range between 0.2 and 10 μm. These researchers discovered a strange phenomenon. The test persons' lungs seemed to function like an “aerosol generator.” Even after minutes of inhaling particle-free air, the subjects exhaled different concentrations of very small aerosol particles. Particles with an aerodynamic size range >5 μm were not found in the exhaled air during quiet breathing.

The team began to investigate this “disruptive factor” in more detail and discovered the following phenomena:

(1) The lungs produce aerosol particles with a size of ∼0.4 μm in diameter.
(2) The production rate differs considerably between individual test persons. The measured aerosol concentrations varied between a few tens of particles per liter of exhaled air to several thousand particles per liter.
(3) The particles are produced during inhalation and are released with the subsequent exhalation.^a^
(4) The particles do not arise in the upper but in the lower very small airways. At the beginning of an exhalation in the first ∼200 mL, there are no or very few particles, and at the end of the exhalation the concentration increases.
(5) Respiratory flow rate had no influence on aerosol concentration.
(6) On test day 1, a multiple of the exhaled particles were measured in one of the subjects. The following day, he reported sick due to a respiratory infection. When he recovered, his aerosol concentration was back to normal.

The hypothesis of how these aerosol particles are created is that small airways collapse during exhalation and are reopened during the subsequent inhalation, creating small mucus/surfactant droplets that are exhaled with the subsequent exhalation. In patients with respiratory infections, the increased production of surfactant and mucus could enhance this process. The data at that time were only published as lectures and posters at a congress.^(2)^

The results of this study and the study itself have been forgotten.

### Inhaling to mitigate exhaled bioaerosols

It was not until 2003 that Prof. David Edwards from Harvard University in Boston contacted Dr. Gerhard Scheuch as a member of the 1987 working group, seeking technical support for an investigation into the spread of influenza viruses from the respiratory tract through breathing and coughing. The online measurement of very small aerosol particles in the nanometer range is technically very difficult and so a special measurement setup was developed to carry out the investigations. The instrument used was able to detect aerosol particles >0.086 μm.

Prof. Edwards and the research group of Dr. Scheuch then carried out a series of investigations, which were published in November 2004 in the Proceedings of the National Academy of Science.^(3)^

Edward's hypothesis was that the transmission of influenza virus occurs by coughing or breathing, and by making the mucus and surfactant (respiratory surfactant) in the upper and lower airways more viscous, the spread of viruses can be reduced. He wanted to use surfactant inhalation to reduce the number of exhaled particles. The study was supported by the Technical Support Working Group of the U.S. Government with the aim of reducing the spread of viruses in American military barracks.

The study showed that the inhalation of surfactant could not minimize the exhalation of aerosol particles, but the opposite occurred. In fact, significantly more exhaled particles were produced (>300%). By contrast, the inhalation of isotonic NaCl (saline) solution succeeded in reducing the exhalation of particles by >70%.

Prof. Edwards continued the study in the United States and found influenza viruses in the exhaled air,^(4)^ which confirmed our hypothesis that the lungs are not only an aerosol generator, but can even be a “virus spreader.”

The exhalation of aerosol particles produced in the lungs has now been confirmed by several other working groups. These researchers also found a particle size of the exhaled aerosol particles in the so-called accumulation mode to be 0.1–0.5 μm. The group led by J. Hohlfeld and K. Schwarz found aerosol particles with a size of 0.3 μm in the exhaled air.^(5–7)^ They used a condensation nucleus counter and a laser photometer and could in that way measure particles >100 nm. They measured with different breathing patterns without coughing. Johnson and Morawska confirmed the production of the aerosol particles in the very small airways, but were only able to determine that the particles had to be significantly smaller than 1 μm because their measuring technology could only detect particles >0.5 μm.^(8)^ They called the mechanism bronchiole fluid film burst, that is, the bursting of the smallest surfactant bubbles in the small airways or the reopening of closed small airways. The “reopening of small airways” is also mentioned by Bake et al. in their various publications as a mechanism for the aerosols produced in the exhaled air.^(9–12)^ In a study in which they carried out a standardized breathing maneuver with all subjects (exhalation to the residual volume before maximum inhalation and measurement in the following complete exhalation), they found an average of ∼10,000 particles/L.^(9)^ This is significantly more than that by Gebhart, Schwarz, and Edwards, who found a wide range of variation, but on average usually significantly fewer particles in the exhaled air. The reason is the standardized breathing maneuver with the deep exhalation. Bake et al. could not determine the particle size exactly, their measuring device could only measure particles >0.41 μm.

In a study by Hersen et al.,^(13)^ it was found that patients with respiratory infections produced a multiple of aerosols (especially in the range of very small aerosol particles <1 μm) than healthy subjects. The group examined patients with influenza and corona infections. With their method they could only determine aerosol concentrations and particle sizes but could not determine whether viruses were also present in these exhaled aerosols. They could measure particles in the size range between 7 nm and 10 μm. In the group of healthy individuals no particles >2 μm could be detected. And even in the patients group the majority of particles (>99%) were found in the size range <2 μm. This study is interesting because it shows that, especially in the particle size range <0.6 μm, a much larger aerosol concentration was found in the infected than in the noninfected patients. The study did support the earlier Gebhart finding with the single subject who had a respiratory infection. In this study, patients were also asked to cough, which of course strongly influences the results.

### The spread of influenza and corona viruses through breathing

Already in 2008, the group led by Patricia Fabian and Donald Milton was able to detect influenza viruses in exhaled aerosol particles on the suggestion of David Edwards. The authors found that 87% of the exhaled aerosol particles were <1 μm in size.^(4)^

Milton et al. again detected influenza viruses in the exhaled air of infected patients.^(14)^ They distinguished between larger aerosol particles >5 μm generated by coughing and smaller aerosol particles <5 μm. In 35 of 37 patients with influenza, they found significant amounts of influenza viruses in the small aerosol range, which were caused by normal breathing, whereas they could only detect virus RNA when coughing in 16 out of 37 patients, and the amounts of virus material collected were also much lower than those found in the small aerosol particles during normal breathing. The group also tested whether breathing masks used by the patients could effectively hold back these particles to protect health care workers. This worked quite well for the coarse aerosol particle fraction, because virus material was only found in 4 out of 37 patients when the patient wore surgical masks. This was not the case for the fine aerosol particle fraction. Viruses were found in 29 of the 37 patients even with a breathing mask. The number of exhaled viruses was reduced by 55% by wearing a surgical mask. Leung et al.^(15)^ also found viruses in the exhaled aerosol particles. They distinguished between particles <5 μm and particles >5 μm. They concluded, “Our findings indicate that surgical masks can efficaciously reduce the emission of influenza virus particles into the environment in respiratory droplets, but not in aerosols.”

Lindsley et al.^(16)^ also found significant amounts of influenza A virus in the exhalate. The authors found slightly more viruses in coughing than in normal exhalation. However, they noted that, of course, coughing occurs much less frequently than breathing and, therefore, the spread of the viruses probably occurs much more frequently and effective through normal breathing.

Fabian et al. also found rhinoviruses in the exhaled particles in infected patients, which were also mainly in the smallest particles that could be measured.^(17)^

The fact that the spread of different viruses occurs through normal breathing of infected persons has now also been proven by various other working groups. Wang et al.^(18)^ speculated that SARS viruses can be transmitted from person to person through aerosol.

Gralton et al.^(19)^ found in 53 patients with various respiratory infections in 80% of infected virus RNA after normal breathing by analyzing air samples in a cascade impactor. The same virus families were also found by Mitchell et al.^(20)^ in exhalation filters from spirometry devices [rhinovirus, respiratory syncytial virus (RSV), influenza A, influenza B, parainfluenza viruses 1, 2, and 3, and human metapneumovirus].

Yip et al.^(21)^ found the RNA of influenza A viruses in aerosol samples in a hospital room. They used a sampler that distinguishes three size fractions of the aerosol (<4, 1–4, and >4 μm). At a distance of 1 m from the patient's bed, he found influenza RNA in all three fractions. In some patients, RNA could even be detected in the corridor in front of the room, but only in the fraction of the smallest particles.

Shiu et al.^(22)^ found significant amounts of influenza A RNA in the aerosol in the ambient air in a children's ward in a patient's room. Influenza RNA was detected in all three observed aerosol fractions (<4, 1–4, and >4 μm). In patients with detected influenza B, this was only the case in 20% of the patients.

It is highly probable that these results can also be applied to the SARS-CoV-2. There are various observations that support this. Bae et al.^(23)^ examined four COVID-19 patients who should cough through two different filters (surgical and cotton filter) or without a filter. At a distance of 20 cm from the mouth, viruses were then collected in a petri dish. There were no significant differences as to whether or not the patient was wearing a face mask. The authors conclude that the particles that carry the viruses are so small that they cannot be retained sufficiently by the mask material. Interestingly, more virus RNA was found on the outside of the masks than on the inside, which suggests that the very large mucus droplets produced when coughing did not contain very many viruses, but that very small aerosol particles that were not thrown against inside of the mask by the impact forces of the air flow and thus were not eliminated by it, were able to escape to the outside and were deposited on the outside of the mask due to poststenotic turbulence.

van Doremalen et al.^(24)^ showed that SARS-CoV-2 can remain airborne in a room for several hours.

In a study from China,^(25)^ a transmission of SARS-CoV-2 in restaurants was reported. The authors demonstrated that airborne transmission by the ventilation system was responsible for these infections. This can only be explained if aerosol particles are small enough to be transported by the air over a certain distance.

In another unpublished observation (Indoor transmission of SARS-CoV-2), Quian et al. found that the corona COVID-19 infection is an “indoor phenomenon” and almost no infections occur outside, that is, outside closed rooms. Of >7000 observed and documented infections, only 1 infection occurred outdoors.

Liu et al. did an aerodynamic analysis of room air at different Wuhan hospitals.^(26)^ In one protective apparel removing room, they found significant amounts of SARS-CoV-2 in the size range 0.25 μm–0.5 μm. They were able to detect particles between few nanometers and 10 μm.

Morawska and Cao^(27)^ point to the many observations that make it extremely plausible that the SARS-CoV-2 epidemic is also influenced, at least to a large extent, by the transmission of exhaled aerosolized viruses, and this must be taken into account to contain the pandemic.

The SARS-CoV-2 have a size of between 60 and 160 nm,^(28)^ which is very similar to the size of influenza viruses (80–100 nm).^(29)^ Therefore, one exhaled breath aerosol particle (which is between 0.1 and 0.5 μm) could possibly contain at least one virus. The number of SARS-CoV-2 to cause an infection is unknown so far. Nikitin et al. reported that for influenza it is estimated that between 300 and 3000 viral copies can cause an infection through the inhaled route.^(30)^

### Exhaled aerosol accumulation mode

In many studies already mentioned, an estimation of the exhaled aerosol particles size was published. In most cases, the measured particles are smaller than the detection limit of the measuring systems used. By coughing, sneezing, speaking, and singing, also much larger particles are generated.^(31–33)^ These particles may also contain viruses, but this is not the contention of this article. The aerosol particle size range that is mainly generated by normal breathing (0.1–0.5 μm) is particularly common in the ambient air. Aerosol physics can explain this fact. There are only a few mechanisms that cause aerosol particles to be eliminated from any room. For very small aerosol particles, this is the mechanism of Brownian molecular motion or diffusion. This mechanism works relatively effectively in the range between 5 and 100 nm. The other important physical mechanism to eliminate particles from room air is by gravity that results in the sedimentation of particles. This mechanism is effective for aerosol particles above ∼0.5–1 μm. Table 1 gives settling velocity for different particle sizes in 20°C room air. Finally, there is separation by impaction, particles cannot follow the air stream and are eliminated from the air flow by inertial forces. This mechanism is only effective above ∼1 μm. For electrically charged particles, there is also separation by electrostatics, which, however, probably does not play a major role in the ambient air and for exhaled particles.

**Table 1.** Settling Velocity and Settling Distance of Aerosol Particles with a Density of 1 g/cm^3^ in Room Air at 20°C

Particle size (μm)	Settling velocity (μm/second)	Distance settled in 1 minute
0.2	1.2	72 μm
0.5	7.5	0.5 mm
1	30	1.8 mm
2	119	7.2 mm
5	746	44.8 mm
10	2985	180 mm
20	11,942	717 mm

This shows that aerosol particles between ∼0.1 and 0.5 μm are not very effectively filtered out of the surrounding air by any physical mechanism. When particles of any size are produced, over time the smallest and largest particles disappear. The particles between 0.1 and 0.5 μm are accumulated in the air, which is the accumulation mode.

And it is precisely the range of the exhaled particles produced by normal breathing. Their physical properties will keep them in the ambient air for a very long time. These particles can remain in room air for many hours if the air in the room is not exchanged or cleaned.

The next difficulty is that, as already mentioned, filtering of these particles is also extremely difficult. They cannot even be deposited very effectively in our respiratory tract. The deposition of inhaled 0.1–0.5 μm particles is only ∼30%.^(34,35)^ That means 70% of the inhaled particles are exhaled again. While deposition occurs to a small extent throughout the entire respiratory tract (nose, mouth, throat, bronchi, bronchioli, and alveoli), the preferred site of deposition for these particles is the peripheral area of the lungs, bronchioli, and alveoli.^(34)^

Simple filter materials that are used in conventional surgical masks can hardly contribute to the separation of these aerosol particles.

What can be done to contain these exhaled viruses?

(1) Outdoor, these particles are very strongly diluted by the open air and will hardly be able to infect other people.
(2) Indoors one should provide for a large air exchange. This measure is classified as very effective.^(36,37)^
(3) High-efficiency particulate air (HEPA) filter systems^(38)^ as well as electric air ionizing systems^(39)^ can also contribute to an increased air cleaning.^b^
(4) Masks have to be developed that provide adequate protection for the clinical personnel working with infectious patients. The current protection appears to be inadequate in many hospital settings.

In conclusion, I would like to state explicitly that I do not believe that normal breathing is the only mechanism that promotes the spread of the SARS-CoV-2. It is likely that the disease spreads through different infection routes. However, exhalation of viruses by breathing of an infected person and subsequent inhalation from the surrounding air by others is an important mechanism and must be considered when containing such a pandemic.

It is imperative that masks with appropriate filtering be developed and produced for health care workers to protect them from viral infection. And in addition efficient filter systems to clean the room air and ventilation to exchange air could be helpful to reduce the transmission.

## Notes

^a^ This was determined by various breathing maneuvers. The production of aerosol particles is increased when the test person starts the breathing maneuver with an inhalation after a very deep exhalation and after a pause in breathing after an exhalation, the exhaled aerosol particle concentration decreases.

^b^ Such ionization separators can charge the very small particles with ions and then contribute to the cleaning of the air by electrostatic separation.

## Notes

Reviewed by:

Timothy Corcoran
